# Computational Treatment Simulations to Assess the Need for Personalized Tamoxifen Dosing in Breast Cancer Patients of Different Biogeographical Groups

**DOI:** 10.3390/cancers13102432

**Published:** 2021-05-18

**Authors:** Anna Mueller-Schoell, Robin Michelet, Lena Klopp-Schulze, Madelé van Dyk, Thomas E. Mürdter, Matthias Schwab, Markus Joerger, Wilhelm Huisinga, Gerd Mikus, Charlotte Kloft

**Affiliations:** 1 Department of Clinical Pharmacy and Biochemistry, Institute of Pharmacy, Freie Universitaet Berlin, 12169 Berlin, Germany; anna.mueller-schoell@fu-berlin.de (A.M.-S.); robin.michelet@fu-berlin.de (R.M.); lena.klopp-schulze@fu-berlin.de (L.K.-S.); gerd.mikus@fu-berlin.de (G.M.); 2 Graduate Research Training Program PharMetrX, 12169 Berlin, Germany; 3 College of Medicine and Public Health, Flinders University, Adelaide, SA 5042, Australia; madele.vandyk@flinders.edu.au; 4 Dr. Margarete Fischer-Bosch Institute of Clinical Pharmacology, Stuttgart, and University Tübingen, 70376 Tübingen, Germany; Thomas.Muerdter@ikp-stuttgart.de; 5 Dr. Margarete Fischer-Bosch Institute of Clinical Pharmacology, 70376 Stuttgart, Germany; Matthias.Schwab@ikp-stuttgart.de; 6 German Cancer Consortium (DKTK), Partner Site Tübingen, German Cancer Research, 69120 Heidelberg, Germany; 7 Departments of Clinical Pharmacology, Pharmacy and Biochemistry, University Tübingen, 72076 Tübingen, Germany; 8 Department of Medical Oncology and Hematology, Cantonal Hospital, 9007 St. Gallen, Switzerland; Markus.Joerger@kssg.ch; 9 Institute of Mathematics, University of Potsdam, 14476 Potsdam, Germany; huisinga@uni-potsdam.de; 10 Department of Clinical Pharmacology and Pharmacoepidemiology, University Hospital Heidelberg, 69120 Heidelberg, Germany; 11 Department of Clinical Pharmacy, Institute of Pharmacy, University of Bonn, 53113 Bonn, Germany

**Keywords:** tamoxifen, breast cancer, CYP2D6, polymorphism, genotype, individualized dosing, personalized dosing, model-informed precision dosing

## Abstract

**Simple Summary:**

Tamoxifen is a drug often used to treat the most common type of breast cancer. Its metabolite endoxifen is formed by the liver enzyme CYP2D6, whose activity is variable and depends on a patient’s genetic profile. The frequency of CYP2D6 variants with different functional enzymatic activity varies largely between populations. To ensure sufficient efficacy of tamoxifen, a certain target concentration of endoxifen is needed, and 20% of White breast cancer patients have been shown not to reach this target concentration. However, little is known about the risk of not attaining the endoxifen target amongst other ethnic populations. This study investigated the risk for suboptimal endoxifen concentration in nine different biogeographical populations based on their distinct CYP2D6 genetic profile. The variability between the populations was high (up to three-fold), and East Asian breast cancer patients were identified as the population with the highest need for personalized tamoxifen dosing.

**Abstract:**

Tamoxifen is used worldwide to treat estrogen receptor-positive breast cancer. It is extensively metabolized, and minimum steady-state concentrations of its metabolite endoxifen (C_SS,min ENDX_) >5.97 ng/mL have been associated with favorable outcome. Endoxifen formation is mediated by the enzyme CYP2D6, and impaired CYP2D6 function has been associated with lower C_SS,min_ _ENDX_. In the Women’s Healthy Eating and Living (WHEL) study proposing the target concentration, 20% of patients showed subtarget C_SS,min ENDX_ at tamoxifen standard dosing. CYP2D6 allele frequencies vary largely between populations, and as 87% of the patients in the WHEL study were White, little is known about the risk for subtarget C_SS,min ENDX_ in other populations. Applying pharmacokinetic simulations, this study investigated the risk for subtarget C_SS,min ENDX_ at tamoxifen standard dosing and the need for dose individualization in nine different biogeographical groups with distinct CYP2D6 allele frequencies. The high variability in CYP2D6 allele frequencies amongst the biogeographical groups resulted in an up to three-fold difference in the percentages of patients with subtarget C_SS,min ENDX_. Based on their CYP2D6 allele frequencies, East Asian breast cancer patients were identified as the population for which personalized, model-informed precision dosing would be most beneficial (28% of patients with subtarget C_SS,min ENDX_).

## 1. Introduction

Accounting for approximately 11.7% of all cases and 24.5% of cases in women, breast cancer has surpassed lung cancer as the most common cancer worldwide [1]. It is known that 40% to 80% of breast cancers are estrogen receptor-positive (ER+) and thus responsive to endocrine therapy. Due to its affordability and high efficacy [2], as shown in a reduction of breast cancer recurrence by around 30% in the first 15 years of therapy, the selective estrogen receptor modulator tamoxifen is widely used in premenopausal and postmenopausal breast cancer patients [3,4].

Following almost exclusively the current ‘one-dose-fits-all’ approach of 20 mg tamoxifen once daily (QD), high interindividual variability in the concentrations of tamoxifen and its most important, approximately 100-fold more active, metabolite endoxifen [5,6] is observed [7,8,9]. Several factors, including polymorphic metabolizing enzymes such as CYP2D6, CYP2C9, CYP2C19, and UDP-glucuronosyltransferases [6,10], patient age [11,12,13,14,15], patient body weight [9,12], hormonal status [16], and treatment adherence [8,17,18] have been identified as contributors to this variability. Among those factors, CYP2D6 functionality, which determines the extent of endoxifen formation, has been found to explain the highest fraction of variability (26.4–53%) [8,17]. Impaired CYP2D6 functionality has been shown to result in reduced endoxifen concentrations and worse clinical outcome [8,9,19]. In the Women’s Healthy Eating and Living (WHEL) study [9], attaining a target therapeutic endoxifen minimum concentration at steady state (C_SS,min ENDX_) of at least 5.97 ng/mL was associated with a 26% lower breast cancer recurrence rate, compared with patients with C_SS,min ENDX_ below this threshold. A similar independent study in pre-menopausal patients later confirmed this target threshold [8]. In the WHEL study, approximately 20% of patients showed C_SS,min ENDX_ below the proposed target and were thus at risk for a non-favorable disease outcome.

The frequency of different CYP2D6 alleles varies widely across the world and amongst populations [20], directly impacting the estimated frequency of breast cancer patients with subtarget C_SS,min ENDX_. As all patients in the WHEL study were from the United States and 87% were of White, non-Hispanic ethnicity, the estimate of 20% of patients at risk for subtarget C_SS,min ENDX_ at tamoxifen standard dosing might not be indicative for breast cancer patients of other populations. Studies on the frequencies and effects of specific CYP2D6 polymorphisms have been performed in other breast cancer populations such as East [21,22,23,24,25,26] and Central/South Asians [27]; however, these studies did not report overall frequencies of patients with subtarget C_SS,min ENDX_.

Given the high relevance of tamoxifen for breast cancer patients worldwide, this work assessed the risk for subtarget C_SS,min ENDX_ in patients of different biogeographical groups [28] at tamoxifen standard dosing based on their respective frequency of different CYP2D6 alleles [29] by applying pharmacometric modelling and simulation. For this, tamoxifen and endoxifen concentrations and demographic data of 1388 patients previously pooled in a large international clinical study database were used to develop a joint parent-metabolite pharmacokinetic model of tamoxifen and endoxifen [12]. This model describes not only the typical concentration–time profile but also several layers of variability around it. Once a reliable model has been developed, model-based simulations are a powerful tool to explore yet unstudied scenarios, such as differences in bioavailability, adherence, or sampling times [30,31]. The aim of this study was to apply the model to virtual breast cancer populations capturing the respective CYP2D6 allele frequencies, simulate tamoxifen standard dosing for each biogeographical group, and determine the frequency of patients with subtarget C_SS,min ENDX_.

We concluded that the biogeographical group has an up to three-fold impact on the percentage of subtarget C_SS,min ENDX_ and that East Asian breast cancer patients are at the highest risk for subtarget endoxifen concentrations. Given the high interindividual variability, a personalized model-informed precision dosing approach for tamoxifen is needed to assure reaching the target C_SS,min ENDX_ in all breast cancer patients.

## 2. Materials and Methods

### 2.1. Virtual Patients of Different Biogeographical Groups

Following the recommendation to use standardized biogeographical groups to represent the global distribution of pharmacogenetic allele frequencies [28], nine virtual populations were generated representing the following groups according to Huddart et al. [28]: American, Central/South Asian, East Asian, European, Near Eastern, Oceanian, Sub-Saharan African, African American/African-Caribbean, and Latino. These groups were based on large-scale sequencing and defined using global autosomal genetic structure [28]. While the African American/Afro-Caribbean and the Latino group are admixed groups characterized by distinct genetic profiles resulting from significant post-colonization and post-Diaspora gene flows, the other groups are based on the geographical distribution of pre-colonization and pre-Diaspora common genetic ancestry [32]. For more information on the grouping system and the populations within each group, please refer to [28,32] and [29], respectively.

Over 100 different CYP2D6 alleles have been identified, whose functional activity is commonly described using the CYP2D6 activity score (AS) system [33,34]. Based on their activity, CYP2D6 alleles are assigned an AS of 0 (no activity), 0.5 (impaired activity), or 1 (full activity). Recently, an AS of 0.25 was assigned to the allele CYP2D6*10 [35]. To obtain the CYP2D6 diplotype AS, each allele is assigned an AS, and the sum is taken. Thus, the CYP2D6 diplotype AS can range from 0 (no activity), through 2 (two fully functional alleles), up to ≥6 (due to copy number variations) [33,34,35].

To allow the generation of a sufficient number of virtual breast cancer patients even with rare CYP2D6 AS, every virtual population consisted of 10,000 patients. Based on the three covariates used in the pharmacokinetic model [12], each virtual patient was assigned a CYP2D6 AS, an age value, and a body weight value. The “Phenotype frequency by group” sheet in the CYP2D6 frequency table of the Clinical Pharmacogenetics Consortium (CPIC) [29] based on ‘real-world’ patient data was used to assign CYP2D6 AS frequencies in the virtual populations and mirror each biogeographical group. Because the classification of CYP2D6*10 as AS 0.25 was recent and thus a covariate for AS 0.25 was not available for use in our pharmacokinetic model, the frequencies of each 0.25 step provided in the CYP2D6 frequency table were assigned to the respective following 0.5 step. Following common practice, patients with missing AS information were assigned the wildtype CYP2D6, i.e., an AS of 2 [29]. If the sum of all AS frequencies per biogeographic group (including the ‘missing AS’ classification) was less than 1, the remaining difference was considered missing and assigned an AS of 2 as well. Frequencies of AS of 2.5 up until ≥6, resulting from copy number variations, were summed and classified as AS of >2. To ease the interpretation, CYP2D6 genotypes were also translated into genotype-predicted phenotypes using the most recent consensus on CYP2D6 genotype-to-phenotype translation [35]: (i) AS of 0 were assigned to genotype-predicted poor metabolizers (gPM), and (ii) AS of 0.5–1 were assigned to genotype-predicted intermediate metabolizers (gIM). AS of ≥1.5 were assigned to genotype-predicted normal metabolizers (gNM; including ultrarapid metabolizers). Age and body weight values were randomly and independently sampled, with replacement from the age (Median: 55 years, range: 22–95 years) and body weight (Median: 67 kg, range: 39–150 kg) values of 1388 patients used for the development of our pharmacokinetic model [12].

### 2.2. Joint Parent–Metabolite Pharmacokinetic Model of Tamoxifen and Endoxifen and Simulations

For all simulations, a previously published nonlinear mixed-effects joint parent–metabolite model of tamoxifen and endoxifen [12] with its final parameter estimates was used. The model consisted of a gut compartment and central compartments for tamoxifen with apparent volume of distribution V_TAM_/F and for endoxifen with apparent volume of distribution V_ENDX_/F. Tamoxifen was described to be absorbed in a first-order process with lag time (characterized by the parameters k_a_ and t_lag_, respectively). Tamoxifen in the central compartment was described to be either metabolized to endoxifen with apparent formation CL_23_/F or to be eliminated via other pathways with apparent clearance CL_20_/F, both linear processes. Upon formation, endoxifen was described to be eliminated in a linear process as well (CL_30_/F). Covariate relationships were implemented on both CL_20_/F (age and body weight, both using power functions) and CL_23_/F (CYP2D6 AS, using fractional change models with reference value AS 2). More specifically, fractional changes in CL_23_/F compared to a CYP2D6 AS of 2 were estimated for ASs 0, 0.5, 1.5, and >2. Interindividual variability parameters were implemented in both tamoxifen clearance pathways, and a log-transformed-both-sides approach was used to characterize the residual variability. Detailed descriptions of model development and covariate selection have been published elsewhere [11,12].

Six months of tamoxifen standard treatment (20 mg QD), assuring endoxifen steady-state attainment, were simulated for each virtual population using NONMEM v. 7.4, called through PsN v. 3.6.2 via Pirana v. 2.9.7 [36]. Next, the percentage of breast cancer patients in each virtual population with C_SS,min ENDX_ below the suggested target threshold of 5.97 ng/mL was assessed, and the risks compared. Pre- and postprocessing was performed in R v.3.5.1 using RStudio Version 1.2.1184, using the packages *dplyr, ggplot2, gridExtra, plyr, Xpose4,* and *zoo*.

## 3. Results

### 3.1. Virtual Patients of Different Biogeographical Groups

The CYP2D6 allele frequency distributions were derived for the nine biogeographical groups [28] as described above. For each biogeographical group, the respective subpopulations in which CYP2D6 genotyping studies had been performed [29] are provided as examples:

- American: e.g., Alaska Natives, American Native Indians, Argentinians, Canadians (Inuit and Native Indians), Chileans (Mapuches), Costa Ricans (Amerindians), Ecuadorians, Mexicans (Amerindians and Mexican Natives), Native Americans from South, North, and Central America as well as Panamanian, Paraguayan, and Venezuelan populations
- African American/Afro-Caribbean: e.g., African American, Antillean, Costa Rican, Cuban, Trinidadian
- Central/South Asian: e.g., Indian, Pakistani, Tamil, and Trinidadian populations
- East Asian: e.g., Chinese, Filipinos, Japanese, Karen, Korean, Malay, Mongolian, Russian (Russian Far East), Thai, Tibetan, Uyghur, and Vietnamese populations
- European: e.g., Albanian, Austrian, Belgian, Brazilian (of European descent), Caucasian, Croatian, Cuban (of European descent), Czech, Danish, Dutch, Faroese, Finnish, French, German, Greek, Hungarian, Italian, Macedonian, North American, Norwegian, Polish, Portuguese, Roma, Russian (Voronezh Region + St. Petersburg), Sardinian, Spanish, Swedish, and Swiss populations
- Latino: e.g., including Admixed Latin American, Hispanic American, Brazilian, Chilean, Columbian, Costa Rican, Cuban, Ecuadorian (Mestizos), Mexican, Nicaraguan, Puerto Rican, and Venezuelan populations
- Near Eastern: e.g., Ashkenazi Jews, Bedouin, Emirati, Iranian, Iraqi, Middle-Eastern, Saudi Arabian, Syrian, and Turkish populations
- Oceanian: e.g., Australian Aborigines, Maori, Papua New Guinean, Hawaiian, Melanesian, and Polynesian populations
- Sub-Saharan African: e.g., Brazilian (with African descent), Ethiopian, Ghanaian, South African, Tanzanian, Xhosa, Venda, Zimbabwean, and Kenyan populations.

**Figure 1:**
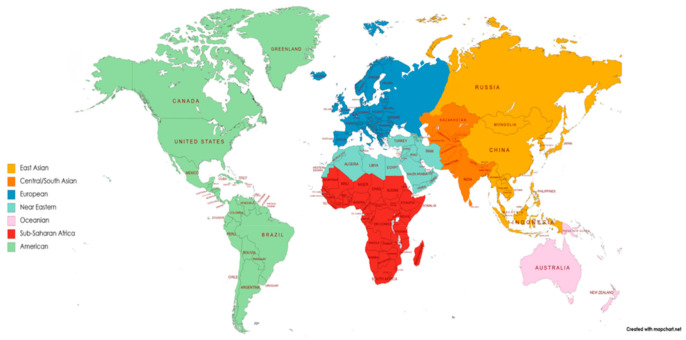
Populations included in the respective biogeographical groups previously defined [28]. As in [28], African American/Afro-Caribbean and Latino groups are not shown in this figure, as the map gives an overview of the genetic ancestor-based borders of geographic groups, and this concept cannot be applied to the two admixed groups. Figure recreated from [28] using https://mapchart.net/ (accessed on 12 January 2021).

The geographic localizations of the different populations allocated to each biogeographical group are illustrated in Figure 1. The final CYP2D6 AS frequencies assigned to each virtual population are provided in Table 1.

CYP2D6 AS frequencies and resulting enzyme functionalities varied largely amongst biogeographical populations. Except for African American/Afro-Caribbeans, East Asians, and Sub-Saharan Africans (AS 1.5 most common), AS 2 was the most common AS for all biogeographical groups with frequencies ranging from 18.6% to 63.7%. While the frequency of AS 0 was highest in Europeans (6.47%), the frequency of AS of ≥2 was highest in Oceanians (20.6%). For all populations, the frequency of missing CYP2D6 AS assigned to AS 2 was low (≤7.41%).

**Figure 2:**
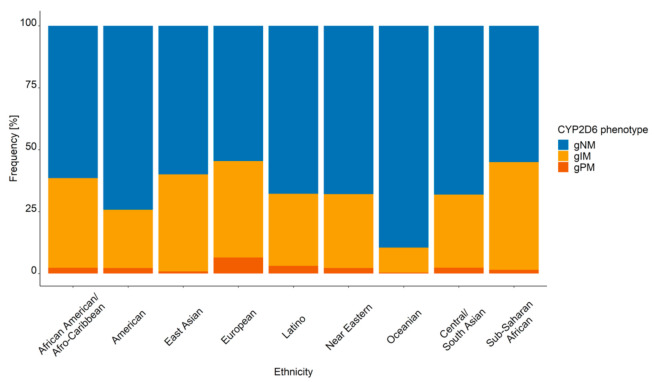
CYP2D6 genotype-predicted phenotype frequencies in the nine virtual populations (*n* = 10,000 patients) representing the biogeographical groups defined in [28]. gNM, gIM, and gPM: genotype-predicted normal, intermediate, and poor metabolizers, respectively.

Resulting from the large differences in the CYP2D6 AS frequencies amongst the biogeographical groups, the frequencies of genotype-predicted phenotypes varied accordingly (Figure 2).

gNM was the most frequent genotype-predicted phenotype in all biogeographical groups. The largest percentages of gNM were encountered in Oceanians (89.5%), Americans (74.2%), Central/South Asians (68.1%), and Near Easterns (68.0%). The highest occurrence of gIM was in Sub-Saharan Africans (43.5%), East Asians (39.2%), and African American/Afro-Caribbeans (36.2%). The highest occurrence of gPM was in Europeans (6.47%).

### 3.2. Simulated Endoxifen Steady-State Concentrations at Tamoxifen Standard Dosing for Each Biogeographic Group

**Figure 3:**
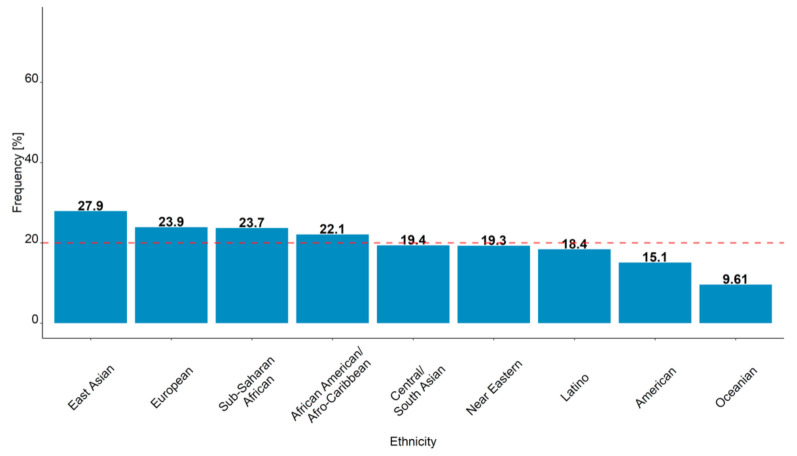
Frequencies of breast cancer patients not attaining C_SS min, ENDX_ upon six months of simulated tamoxifen standard dosing in the nine virtual populations (*n* = 10,000 patients, each) representing the biogeographical groups defined in [28]. *Red dashed line*: reported frequency of patients with subtarget C_SS min, ENDX_ in the WHEL study (20%) [9]. C_SS,min ENDX_: Endoxifen minimum concentrations at steady state; WHEL: Women’s healthy eating and living.

The large differences in CYP2D6 allele frequencies translated into a substantial variability in the percentages of patients with subtarget C_SS,min ENDX_ after six months of tamoxifen standard treatment (Figure 3). The percentage of patients not reaching target C_SS,min ENDX_ was lowest in Oceanians, with 9.61% of patients showing subtarget C_SS,min ENDX_. In contrast, East Asians showed an approximately three-fold higher frequency, with 27.6% of virtual patients showing subtarget C_SS,min ENDX_.

In four out of the nine virtual biogeographical groups, more than 20% of patients would not reach the C_SS,min ENDX_ target concentrations as defined in the WHEL study [9]. Similarly, in five out of the nine virtual biogeographical groups, less than 20% of patients would not reach the C_SS,min ENDX_ target concentrations as defined in the WHEL study. The simulated percentage of Europeans with subtarget C_SS,min ENDX_ was comparable with the result of 20% reported for the mostly White patients in the WHEL study. These simulation results emphasize the magnitude of the variability in C_SS,min ENDX_ between different breast cancer populations upon tamoxifen standard dosing and highlight the need for personalized tamoxifen dosing to assure reaching the target C_SS,min ENDX_ in all breast cancer patients.

## 4. Discussion

Tamoxifen is one of the most frequently prescribed drugs for the treatment of estrogen receptor-positive breast cancer worldwide. Despite the high interindividual variability in its exposure and research suggesting an efficacy threshold for its metabolite endoxifen [8,9], a fixed dose of 20 mg tamoxifen QD is usually prescribed. In the WHEL study, 20% of breast cancer patients showed subtarget C_SS,min ENDX_ [9]. However, endoxifen formation largely depends on CYP2D6 functionality, and the WHEL study, despite its large sample size of 1370 participants, could only represent a small part of the global breast cancer population and its associated diversity.

Based on the large differences in CYP2D6 allele frequencies amongst biogeographical patient populations [28,29] and the key role of CYP2D6 in endoxifen formation, this study aimed to investigate the magnitude of variability in endoxifen target attainment among biogeographical patient populations. As expected, the differences in C_SS,min ENDX_ target attainment were significant due to the large variability in CYP2D6 allele frequencies. While only 1 out of 10 Oceanians showed subtarget C_SS,min ENDX_, inadequate concentrations were observed in 1 out of 3 East Asians. Moreover, approximately half of the breast cancer patients in Asia are pre-menopausal [37]. Given that pre-menopause is another risk factor for sub-target C_SS,min ENDX_ [12], personalized tamoxifen dosing will be most beneficial for East Asians breast cancer patients.

The model applied in this work was developed based on a large dataset comprising 1388 patients of 5 different ethnicities (African, Asian, Indians, Middle-Eastern Arab, and White) and was able to capture the observed concentrations across all CYP2D6 activity scores well [12]. Thus, it was suitable to be applied for simulations of the expected endoxifen concentrations based on the most frequent CYP2D6 allele frequencies in the distinct biogeographical groups. Moreover, simulated endoxifen concentrations were well in line with published measured endoxifen concentrations at 20 mg tamoxifen once daily (e.g., median simulated C_SS,min ENDX_ in biogeographical Europeans of 9.50 ng/mL vs. measured (i) mean C_SS,min ENDX_ in 353 US American breast cancer patients of 7.92 ng/mL [38], (ii) median C_SS,min ENDX_ in 42 European breast cancer patients of 8.99 ng/mL [39], and (iii) measured median C_SS,min ENDX_ in 121 mostly European breast cancer patients of 9.64 ng/mL [40]; as well as median simulated C_SS,min ENDX_ in biogeographical Near Easterns of 10.3 ng/mL vs. measured mean C_SS,min ENDX_ in 117 Iranian breast cancer patients of 11.1 ng/mL [41]).

While we previously identified two additional covariates (age and body weight) to be influential on C_SS,min ENDX_ [12], this work solely focused on the expected frequencies of patients with subtarget C_SS,min ENDX_ based on the differences in CYP2D6 allele frequencies in the different biogeographical groups. Undoubtedly, differences in body weight and age distributions exist amongst the biogeographical groups. However, correctly capturing these differences for the biogeographical groups is strongly compromised due to the biogeographical groups being based on the geographical distribution of pre-colonization and pre-Diaspora common genetic ancestry [28]. While this grouping system is very logical from a genetics point of view, populations in a joint biogeographical group are often nowadays located in different geographical regions (for example, the ‘European’ biogeographical group comprises populations from the European, the North-American, the Middle-American, and the South-American continents) [28]. As typical age and body weight distributions often vary between these populations, the same body weight and age values were used for all virtual patient populations to prevent the introduction of bias. Moreover, compared to the CYP2D6 AS, age and body weight contribute much less to the observed interindividual variability in endoxifen formation [12]. Yet, if needed, dedicated treatment simulations using respective body weight and age distributions could be performed for specific populations, such as the citizens of a country. In general, the more influential factors on endoxifen concentrations are included in the simulations, the more difficult it becomes to assess the impact attributable to each factor. As CYP2D6 activity is by far the most influential factor on endoxifen concentrations, we studied the impact of different CYP2D6 allele frequencies on the expected endoxifen target concentration attainment. Similar studies could be performed for other influential factors to assess the impact of their distinct distributions in different patient populations.

A limitation of this study is the classification of individuals into one of nine biogeographical groups, thus simplifying the complexity of CYP2D6 allele frequency distributions worldwide and within a population. Furthermore, the estimated CYP2D6 allele frequencies are based on the combined results of single studies assessing genotypes in distinct populations within a biogeographical group [29]; as such, the reported CYP2D6 allele frequencies are always approximations with variable margins of errors [29]. These margins of error depend on how well the biogeographical groups are represented by the subpopulations studied to estimate the respective biogeographical group CYP2D6 allele frequencies. Furthermore, they depend on how well frequent alleles within a population are studied, i.e., how often these alleles are tested for. Moreover, there is still no consensus on the number of alleles to be tested, which also results in more or less frequent mismatches.

Because of these limitations, the biogeographical groups used in this study should not be used as a substitute for individual CYP2D6 activity assessment. Rather, the biogeographical groups should help to roughly estimate the CYP2D6 functionality-related risk for subtarget C_SS,min ENDX_ in different breast cancer populations and highlight populations with highest need for personalized dosing. 

Despite its potential, routine CYP2D6 genotyping before initiation of tamoxifen therapy, which could guide the selection of an individualized initial dose, is still not established. At the same time, limitations of genotype-guided initial dose selection exist, such as phenocopying by the concomitant use of certain comedications, which cannot be accounted for by genotyping [42]. Consequently, recent research suggests that phenotyping instead of genotyping could be more adequate for predicting the extent of endoxifen formation [43,44,45] and thus for guiding initial tamoxifen dose selection.

In addition, it should be considered that CYP2D6 functionality is only one contributor to the variable endoxifen exposure and that other factors such as polymorphisms of other metabolizing enzymes [6,10], CYP2D6-inhibiting comedication [45], age [11,12,15], body weight [9,12], hormonal status [16], and treatment adherence [8,17,18] should be accounted for as well. Because of the many factors to be considered when predicting the probability of C_SS,min ENDX_ target attainment, we strongly suggest to use a model-informed precision dosing framework [11,46] in the personalized dose-finding process.

It should also be noted that even though several studies have supported the existence of a relationship between CYP2D6 functionality and/or C_SS,min ENDX_ and response to tamoxifen [8,9,19], others have failed to do so [47,48,49]. However, due to differences in study design, patient populations, and DNA source used for CYP2D6 genotyping [7,50], the comparability of all relevant studies is limited. Furthermore, to our knowledge, no prospective study so far had sufficient power to detect the proposed relationships [51,52]. Thus, a well-designed and sufficiently powered [52] prospective clinical trial has to be performed to investigate the discussed relationship between CYP2D6 functionality and/or C_SS,min ENDX_ and tamoxifen response (e.g., overall survival, progression-free survival). This trial should also revisit the endoxifen target threshold concentration proposed in the WHEL study [9] and used in this work (5.97 ng/mL). Alternative endoxifen concentrations, such as 3.36 ng/mL [53] and 5.28 ng/mL [8], have been proposed as efficacy thresholds, and in a recent study in the preventive setting, no differences in response were seen for patients with different endoxifen concentrations receiving 5 mg of tamoxifen once daily [54]. While the low-dose tamoxifen study in the preventive setting was not sufficiently powered to detect the proposed relationship between endoxifen and treatment response, it is possible that different endoxifen target thresholds will be identified for different treatment settings and that there might be no minimum endoxifen target concentration for efficacy in the preventive setting. The suggested trial could also integrate routine CYP2D6 phenotyping using, e.g., a microdosed probe substance, such as yohimbine, and assess the correlation of CYP2D6 phenotype and C_SS,min ENDX_ [55]. The CYP2D6 phenotype could then be used to guide initial dose selection followed by therapeutic drug monitoring-guided dose refinement as part of a previously proposed model-informed precision dosing framework [11,31].

## 5. Conclusions

Despite evidence pointing towards the need for individualized tamoxifen dosing, a fixed dosing approach is still mostly applied. Our treatment simulations for distinct biogeographical groups highlighted the substantial interindividual variability in endoxifen concentrations to be expected based on the different frequencies of CYP2D6 alleles alone. Given that the proposed exposure–response relationship will be confirmed in the future, this study provides a strong argument for personalized, model-informed precision dosing of tamoxifen for all breast cancer patients, especially for biogeographical groups with a high frequency of CYP2D6 alleles with reduced or lost enzyme activity.

## Figures and Tables

**Table 1:** CYP2D6 activity scores frequencies simulated in the nine virtual breast cancer populations (*n* = 10,000 patients) representing the biogeographical groups defined in [28].

Virtual Population	AS 0	AS 0.5	AS 1	AS 1.5	AS 2 ^1^	AS > 2
American	2.18%	1.46%	22.1%	7.24%	61.4%	5.61%
African American/Afro-Caribbean	2.33%	10.5%	25.8%	32.1%	24.7%	4.67%
Central/South Asian	2.34%	7.36%	22.2%	25.8%	39.9%	2.48%
East Asian	0.865%	27.9%	11.2%	36.6%	22.0%	1.38%
European	6.47%	7.52%	31.4%	17.0%	34.5%	3.13%
Latino	3.12%	4.77%	24.3%	17.0%	46.2%	4.58%
Near Eastern	2.20%	8.34%	21.5%	26.7%	30.9%	10.4%
Oceanian	0.383%	0.482%	9.61%	5.22%	63.7%	20.6%
Sub-Saharan African	1.53%	12.3%	31.2%	31.7%	18.6%	4.70%

^1^ The following percentages of CYP2D6 AS were missing and thus assigned ASs of 2: African American/Afro-Caribbean: 1.36%, American: 5.10%, Central/South Asian: 3.77%, East Asian: 7.41%, European: 0.45%, Latino: 4.20%, Near Eastern: 3.75%, Oceanian: 2.59% and Sub-Saharan African: 7.20%. AS: CYP2D6 activity score.

## Data Availability

The simulation datasets presented in the current study are available from the corresponding author upon reasonable request.
